# *Veillonella parvula* acts as a pathobiont promoting the biofilm virulence and cariogenicity of *Streptococcus mutans* in adult severe caries

**DOI:** 10.1128/spectrum.04318-23

**Published:** 2024-09-30

**Authors:** Yuan Wei, Yu Zhang, Yuan Zhuang, Yifei Tang, Hua Nie, Yequan Haung, Ting Liu, Weidong Yang, Fuhua Yan, Yanan Zhu

**Affiliations:** 1 Nanjing Stomatological Hospital, Affiliated Hospital of Medical School, Institute of Stomatology, Nanjing University, Nanjing, China; 2 Department of Endodontics, Nanjing Stomatological Hospital, Affiliated Hospital of Medical School, Institute of Stomatology, Nanjing University, Nanjing, China; 3 Department of General Dentistry, Nanjing Stomatological Hospital, Affiliated Hospital of Medical School, Institute of Stomatology, Nanjing University, Nanjing, China; State Key Laboratory of Food Science and Resources, Nanchang, Jiangxi, China

**Keywords:** adult severe caries, oral microbiota, metagenomic study, *Streptococcus mutans*, *Veillonella parvula*, biofilm, cariogenicity

## Abstract

Adult severe caries (ASC) brings severe oral dysfunction and treatment difficulties to patients, and yet no clear pathogenic mechanism for it has been found. This study is focused on the composition of dental plaque microbiome profiles in order to identify disease-relevant species and to investigate into their interactions with the *S. mutans*. Samples of dental plaque were collected for metagenomic analysis. The acidification, aciduricity, oxidative stress tolerance, and *gtf* (glucosyltransferase) gene expression of *S. mutans* cocultured with *V. parvula* which was identified as ASC-related dominant bacterium. The bioﬁlm formation and extracellular exopolysaccharide (EPS) synthesis of dual-strain were analyzed with scanning electron microscopy (SEM), crystal violet (CV) staining, live/dead bacterial staining, and confocal laser scanning microscopy (CLSM). Furthermore, rodent model experiments were performed to validate the *in vivo* cariogenicity of the dual-species biofilm. The most significantly abundant taxon found associated with ASC was *V. parvula*. *In vitro* experiments found that *V. parvula* can effectively promote *S. mutans* mature biofilm formation with enhanced acid resistance, hydrogen peroxide detoxicity, and biofilm virulence. Rodent model experiments revealed that *V. parvula* was incapable of causing disease on its own, but it significantly heightened the biofilm virulence of *S. mutans* when being co-infected and augmented the progression, quantity, and severity of dental caries. Our findings demonstrated that *V. parvula* may act as a synergistic pathobiont to modulate the metabolic activity, spatial structure, and pathogenicity of biofilms of *S. mutans* in the context of ASC.

**IMPORTANCE:**

Adult severe caries (ASC), as a special type of acute caries, is rarely reported and its worthiness of further study is still in dispute. Yet studies on the etiology of severe caries in adults have not found a clear pathogenic mechanism for it. Knowledge of the oral microbiota is important for the treatment of dental caries. We discovered that the interaction between *V. parvula* and *S. mutans* augments the severity of dental caries *in vivo*, suggesting *V. parvula* may act as a synergistic pathobiont exacerbating biofilm virulence of *S. mutans* in ASC. Our findings may improve the understanding of ASC pathogenesis and are likely to provide a basis for planning appropriate therapeutic strategies.

## INTRODUCTION

Rampant caries, also known as aggressive caries, is characterized by the rapid development of the disease, the appearance of caries on multiple teeth and dental surfaces within 6–12 months, and the propensity to coronal tissue destruction and pulp infection. Thus, there poses a greater difficulty in the treatment process (1). It is mostly reported in children or patients with the systemic diseases such as Sjogren’s syndrome, patients receiving chemoradiotherapy, suffering from salivary gland dysfunction, drug abuse, depression, and AIDS (2–5). When rampant caries occurs in childhood, it is called “bottle caries” or “early childhood caries”; when it occurs in adulthood, Akpata et al. simply call it “adult severe caries” (ASC), whose clinical presentation is rather similar to that of rampant caries, occurring in adult patients without systemic disease (6). However, ASC, as a special type of acute caries, is rarely reported and deserves further study. Yet, studies on the etiology of severe caries in adults have not found a clear pathogenic mechanism.

With the development of modern oral microecology and the introduction of sequencing-based technology and bioinformatics analysis, the understanding of caries microbiology has been greatly enriched in recent years. The caries-causing bacteria have evolved from the traditional single *S. mutans* to oral symbiotic bacteria. Caries occurs due to an ecological imbalance of the oral microbiota, i.e., the growth and reproduction of cariogenic microbiota due to external factors or the disruption of homeostasis by one’s own factors (7). Changes in the oral microbiota can involve many pathological and non-pathological aspects within the oral cavity (8). Although other microbial species (like *Candida albicans*, *Streptococcus anginosus*, and *Selenomonas sputigena*) on tooth surfaces have been associated with dental caries, the pathogenic core microbiota of different types of dental caries is different (9–12), and it remains unclear whether they are active contributors, inactive cohabitants, or they interact with *S. mutans* as pathobionts to promote disease development (13).

Knowledge of the oral microbiota is important for the management of dental caries. However, whether or not the oral microbiota in ASC patients differs is unclear. The microbiology of rampant caries has been an ongoing area of study. But less attention is paid to ASC than to that of the children. As the aggressive caries is the most serious type of dental caries and is difficult to prevent and treat, ASC brings severe oral dysfunctions and treatment difficulties to patients, as well as heavy financial and medical burdens to families and societies. Meanwhile, with more and more studies on the associations between dental caries and systemic diseases (such as diabetes, asthma, gastrointestinal diseases, rheumatoid arthritis, and systemic lupus erythematosus) (14), scholars have found that the severe dental caries induced by *S. mutans* may contribute to the pathogenicity in injured heart tissue (15). A population-based cohort study of Korean adults during 2002–2013 emphasized the relationships between severity of dental caries and coronary heart disease (CHD), suggesting the prevention and clinical treatment of ASC also relate to the prevention of coronary heart disease (16). And clinical studies in an adult cohort displayed a correlation between greater caries and gastrointestinal diseases and systemic lupus erythematosus (17, 18). There is a direct effect of untreated ASC on systemic health and associated quality of life. There is also a clear need for a deeper understanding of the important role of the oral microbiome and its interactions in the development of ASC. Hence, in the current study, we collected supragingival dental plaque from adults with severe dental caries to capture certain bacterial taxa, identified species associated with severe dental caries in adults, and studied associated microbes and their interactions with the major cariogenic bacteria *S. mutans* by *in vitro* and *in vivo* experiments. This is one of the first studies that explored the oral microbiome profiles of adults severe caries and analyzed the disease-associated microbiota and their interactions.

## RESULTS

### Overview of the clinical samples

Thirteen samples were collected from seven patients with ASC (HC group) and six caries-free group (CF group). The mean age of the participants in this study was 25 ± 4.06 years. Table 1 demonstrates the demographics and caries activity of the study subjects.

**TABLE 1:** Demographics and carious characteristics of the study participants

Variable	Caries severity		Value	*P*
	CF (*n* = 6)	HC (*n* = 7)		
Age (years)	26.17 ± 3.06	24.00 ± 4.76	−0.932	0.366
Gender (M/F)	0/6	2/5		0.462
DMFS score	0	56.14 ± 11.78	−3.156	0.001
DMFT score	0	23.71 ± 2.75	−3.175	0.001

### Dental plaque microbial profile analysis of ASC and caries-free subjects

**Fig 1:**
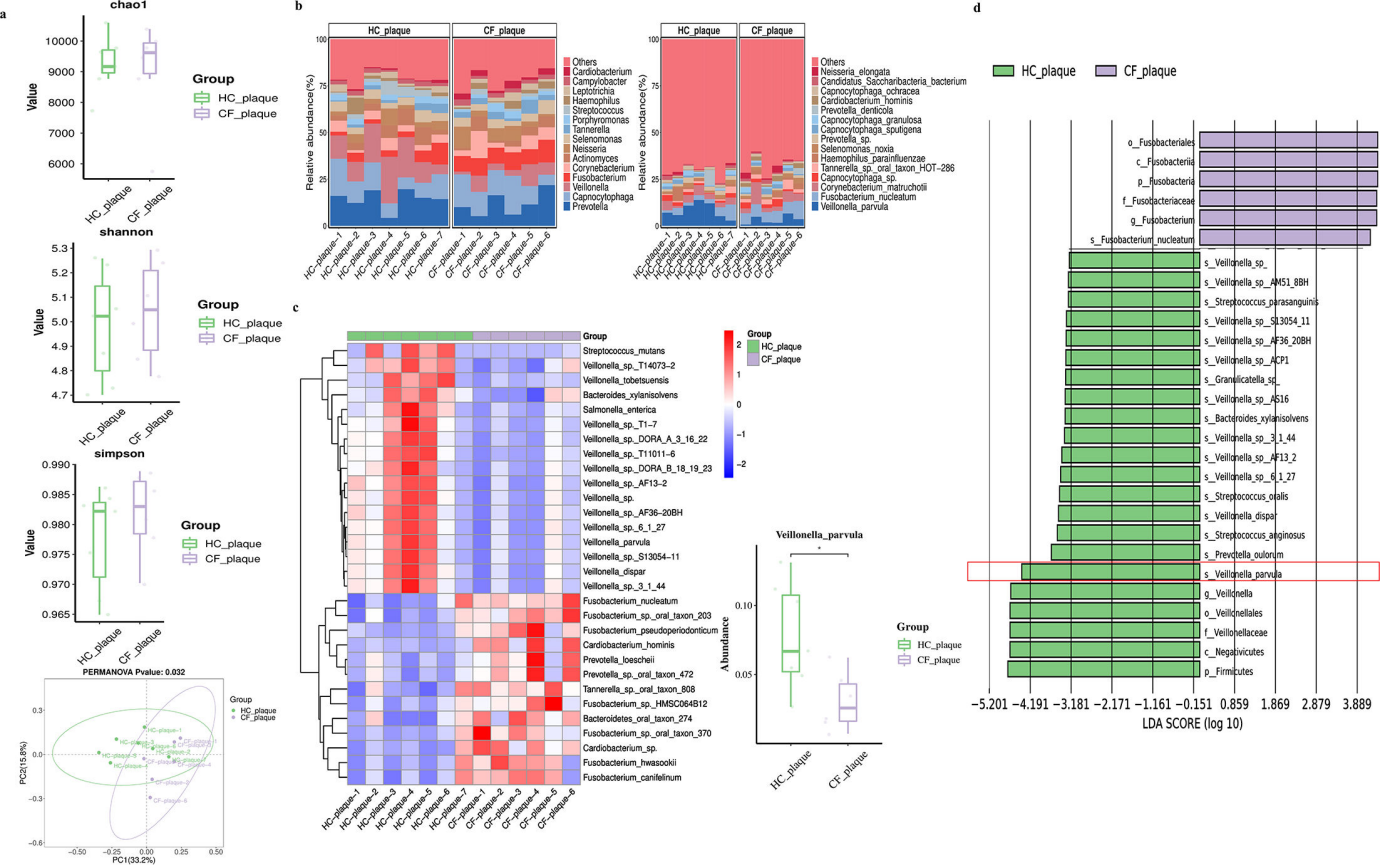
The differences of supragingival dental plaque in patients with adult severe caries and caries frees according to the metagenomic sequencing data. (**a**) Alpha-diversity indices of bacterial species in adult severe caries plaque (HC) and caries frees plaque (CF), including Chao1, Shannon and Simpson index, and Beta-diversity analysis of the plaque microbiota. (**b**) Relative abundance of microbiota at the genus and species levels between two groups. (**c**) Heatmap showing the predominant bacterial at the species level between two groups. (**d**) LDA Effect Size (LEfSe) Cladograms showing differences in the bacterial taxa between HC group and CF group. HC group, adult severe group; CF group, caries-free group.

The species diversity analysis is shown in Fig. 1a. The α-diversity index showed that there was no significant difference in richness and diversity between the HC and CF group. The β-diversity analysis showed the two groups were obviously grouped. The relative abundance of the composition of the two groups of dental plaque is shown in Fig. 1b. At the genus level, the HC group possessed a significantly higher relative abundance of *Streptococcus* spp., *Veillonella* spp. (*P* < 0.05), and *Actinomyces* spp. Specifically, a significant increase in *Prevotella* sp., *Capnocytophaga granulosa,* and *Veillonella parvula* (*P* < 0.05) abundances, as well as a decrease in that of *Fusobacterium nucleatum* (*P* < 0.05) was detected in the HC group at the species level. Notably, from the heatmap, among the 30 most predominant bacterial found in both HC and CF groups at the species level, the relative abundance of *Streptococcus mutans* and *Veillonella parvula* was significantly increased in the HC group versus the CF group (*P* < 0.05) (Fig. 1c). Furthermore, based on the LEfSe results (Fig. 1 d), we found that *V. parvula* was the significantly differentially enriched strain in the HC group, which may be associated with the occurrence and development of ASC.

### Co-culturing improves the acidification, aciduricity, and oxidative stress tolerance of *S. mutans*

**Fig 2:**
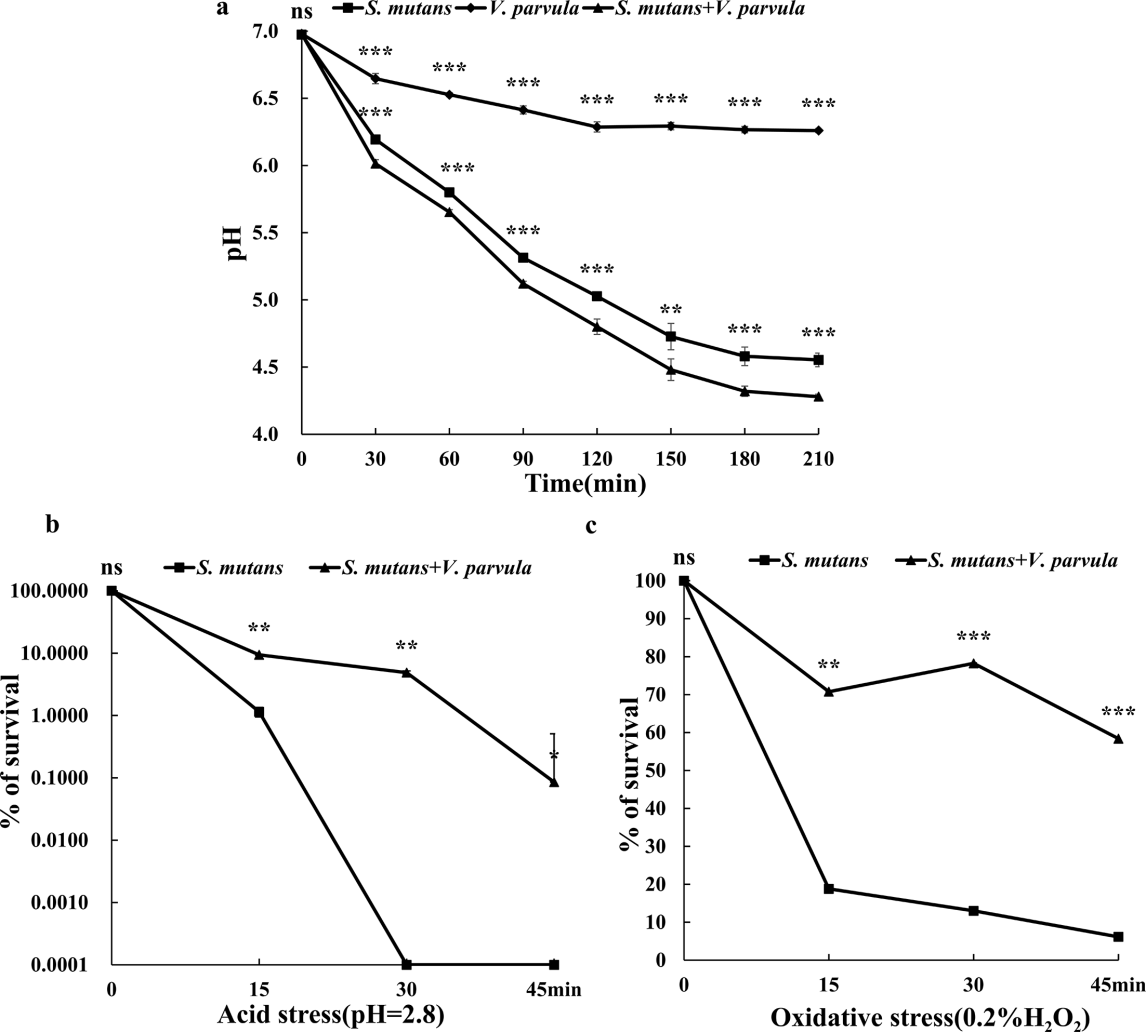
The effect of *V. parvula* on the acidification, aciduricity, and oxygen tolerance of *S. mutans*. (**a**) Glycolytic pH drop. (**b**) The acid tolerance curve. (**c**) The oxygen tolerance curve. The experiments were performed in biological and technical triplicates. All data are presented as the means ± SDs from at least three independent experiments. *P < 0.05, **P < 0.01, and ***P < 0.001.

As metagenomic sequencing results identified *V. parvula* as a potential bacterium associated with the occurrence and development of ASC, we further conducted *in vitro* studies to observe the effect of *V. parvula* on the acidification, aciduricity, and oxidative stress tolerance of the cariogenic strain *S. mutans*. As shown in Fig. 2a, the pH dynamics curve revealed that the pH values of both *S. mutans* and co-culture groups decreased to above 4.0, which could cause enamel demineralization. Meanwhile, the pH value of co-culture system was significantly lower than that of *S. mutans* group during the observation period. To further confirm the effect of commensal state on *S. mutans* aciduricity in a lethal acidic environment, Fig. 2b represented the *S. mutans* survival rate in acid-killing assay. When co-cultured with *V. parvula, S. mutans* displayed a higher survival by over eight folds to the control group (*P* < 0.01) at the 15 min time point. Furthermore, when the reaction time was increased to 30 and 40 min, in which the control group could not survive, on the other hand, *S. mutans* in the co-cultured group still had a survival rate of 4.87% ± 1.79% and 0.09% ±1.15%, respectively (*P* < 0.01), corroborating the improved aciduricity of *S. mutans*. In the hydrogen peroxide killing assay, *S. mutans* also exhibited a higher survival rate (70.8% ± 0.13%, 78.2% ± 0.13%, 58.4% ± 0.15%, respectively) than the control specimen (18.8% ± 0.61%, 13.0% ± 0.61%, 6.16% ± 0.38%, respectively; *P* < 0.01) during the monitoring time in co-cultivation system (Fig. 2c), indicating that oxidative stress tolerance of *S. mutans* were strongly augmented by the commensal.

### Co-culturing of *S. mutans* and *V. parvula* enhances the bioﬁlm formation

**Fig 3:**
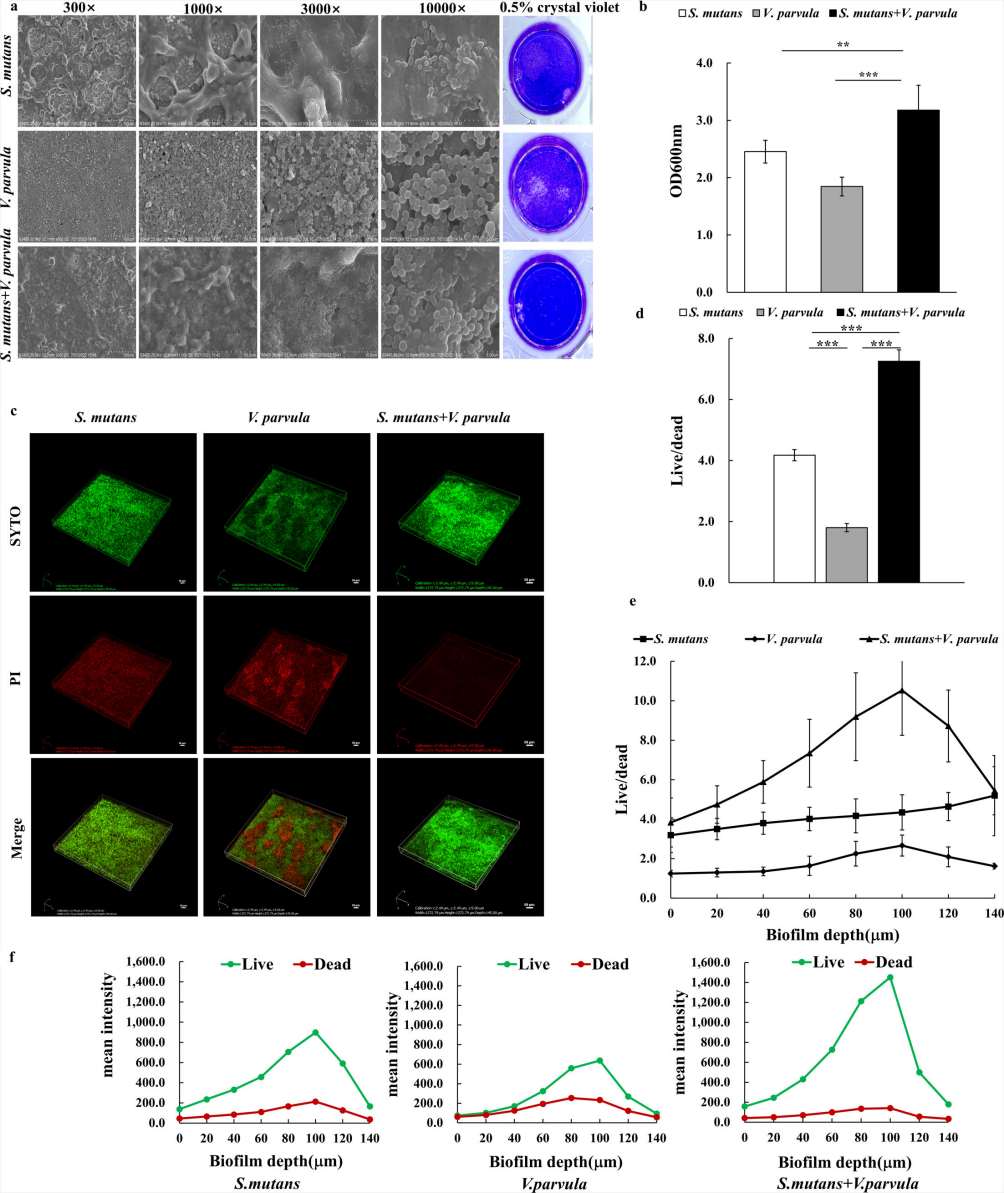
Effect of *V. parvula* on biofilm formation of *Streptococcus mutans*. (**a**) Representative SEM and crystal violet staining images of biofilms. (**b**) Biomass of biofilms quantified with crystal violet staining assay (0.5% crystal violet). (**c**) Representative three-dimensional visualization of live (green) and dead (red) cells in biofilms formed on saliva-coated hydroxyapatite (sHA) discs. (**d**) Integrated ﬂuorescence density of green and red fluorescence in biofilms quantified using software NIS-Elements AR. (**e, f**) Analysis of the average intensity of green and red fluorescence in every 20 µm unit throughout the biofilm; (**e**) the ratio of live and dead bacteria in each layer, (**f**) mean fluorescence intensity of live and dead bacteria in each layer. The experiments were performed in biological and technical triplicates. Values are presented as means ± SDs from at least three independent experiments. *P < 0.05, **P < 0.01, and ***P < 0.001.

Scanning electron microscopy (SEM) observations revealed that *V. parvula* alone was not able to establish a robust biofilm (Fig. 3a). The structure of *V. parvula biofilm* was relatively thinner and more porous, in which the extracellular matrix was invisible. The thickness of the dual-species biofilm increased, and the structure became denser. *V. parvula* aggregated and adhered to the long chain structure of *S. mutans* wrapped by the dense extracelluar matrix, which was similar to the adhesion pattern among *Veillonella* spp. and *Streptococcus gordonii* (19). Subsequently, the crystal violet (CV) staining assay (Fig. 3a and b) indicated that interspecies interactions may significantly promote the colonization of *V. parvula and* the biofilm formation of *S. mutans*, where the biofilm biomass of co-cultivation system (3.178 ± 0.433) was 1.3-fold higher than that of the *S. mutans* group (2.454 ± 0.198, *P* < 0.01).

CLSM images of biofilm were used to visualize the structure of biofilm upon live/dead bacterial staining. Live cells were dyed green, while dead cells were dyed red (Fig. 3c). The strong red ﬂuorescence in the *V. parvula* group embedded in the green viable bacteria indicated a large number of dead cells in the bioﬁlms, which was consistent with the sparse and porous biofilm structure in SEM analysis. Dual-strain bioﬁlms exhibited signiﬁcantly enhanced green ﬂuorescence and less red ﬂuorescence as compared to the *S. mutans* group and the *V. parvula* group, further *indicating* that co-culture obviously promoted the growth of *S. mutans* and *V. parvula*. Quantitative ﬂuorescence intensity (Fig. 3d) conﬁrmed that the live/dead bacterial ratio of dual-species biofilm (7.253 ± 0.369) was signiﬁcantly higher than those of the bioﬁlms formed by the *S. mutans* group (4.177 ± 0.184, *P <* 0.001) and the *V. parvula* group (1.802 ± 0.133, *P* < 0.001), which was consistent with the marked increase of biofilm biomass in the CV staining assay*.* Moreover, the distributions of live cells and dead cells from the surface to the bottom of the selected 140 µm thickness biofilms were quantified using software NIS-Elements AR according to the reported method (20); we found that *S. mutans and V. parvula* exert a remarkable mutually reinforcing effect on bacteria growth when co-cultured. As shown in Fig. 3e and f, the mean ﬂuorescence intensity analysis demonstrated that the live/dead bacterial ratio and the signal of green ﬂuorescence were remarkably higher from the surface (height = 0 µm) to the bottom (height = 140 µm) of the biofilm formed by dual-species when compared with the *S. mutans group* and the *V. parvula* group.

### *V. parvula* enhances the glucosyltransferase activities of *S. mutan*s and boosts *S. mutan*s to produce more EPS

**Fig 4:**
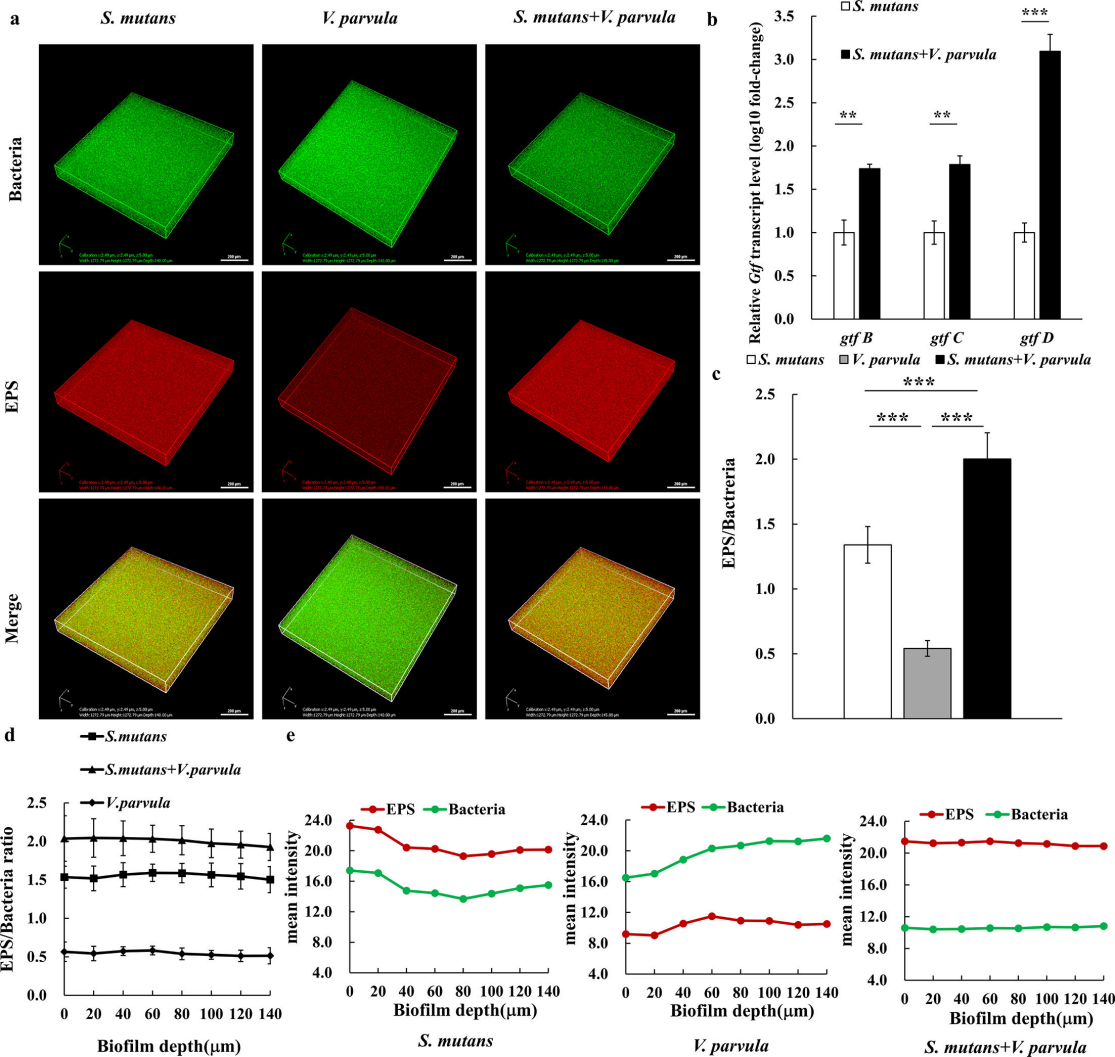
Effect of *V. parvula* on glucosyltransferase activities and exopolysaccharide (EPS) synthesis of *Streptococcus mutans*. (**a**) Confocal laser scanning microscopy (CLSM) images of biofilm EPS synthesis, Green, bacterial cells (stained with SYTO 9); red, EPS (stained with Alexa 647). (**b**) The expression profiles of the *gtf* gene. (**c**) Integrated ﬂuorescence density of EPS and bacteria in the biofilm. (**d, e**) The mean intensity of EPS and bacteria in every 20 µm unit throughout the biofilm; (**d**) the ratio of EPS and bacteria in each layer, (**e**) mean fluorescence intensity of EPS and bacteria in each layer. The experiments were performed in biological and technical triplicates. Values are presented as means ± SDs from at least three independent experiments. *P < 0.05, **P < 0.01, and ***P < 0.001.

*S. mutans* GTFs can metabolise ingested sucrose into glucans, among which water-insoluble glucans can form colloidal EPS as the main component of the biofilm extracellular matrix (21). The expression profiles of *S. mutans* virulence-associated genes after being co-cultured with *V. parvula* were shown in Fig. 4b. Compared with the levels observed in *S. mutans* biofilms, the expression of *S. mutans gtf* genes including *gtfb, gtfc, and gtfd* in dual-strains biofilm dramatically increased by 1.74 times (*P* < 0.01), 1.79 times (*P* < 0.01), and 3.09 times (*P* < 0.001), respectively. The CLSM image confirmed the change in EPS. Fig. 4a showed three-dimensional images of biofilms with green-labeled bacteria and red-labeled EPS. Compared with *S. mutans* mono-species biofilm, the red-stained density of EPS in the co-culture dual-species biofilm was significantly heightened in accordance with the results of SEM. As shown in Fig. 4c, co-culture significantly increased the EPS/bacteria ratio of biofilm, with the ratio being approximately 1.5 times higher than that of the *S. mutans* group (*P* < 0.001). Moreover, the distributions of bacteria and EPS and the EPS/bacterial ratio of the selected 140 µm thickness biofilms were quantified (Fig. 4d and e). As shown in Fig. 4d, the EPS/bacteria ratio in each layer of the biofilm formed by dual-species was significantly higher than those of the biofilms formed by respective mono-strain biofilm. Furthermore, the mean ﬂuorescence intensity analysis (Fig. 4e) revealed that the enhanced EPS generation ability of *S. mutans* in dual-species biofilm, suggesting that this co-culture significantly enhances the EPS production and co-development of structured biofilms, leading to highly cohesive, co-assembled biofilms.

### *V. parvula* promotes the cariogenicity of *S. mutan*s *in vivo*

**Fig 5:**
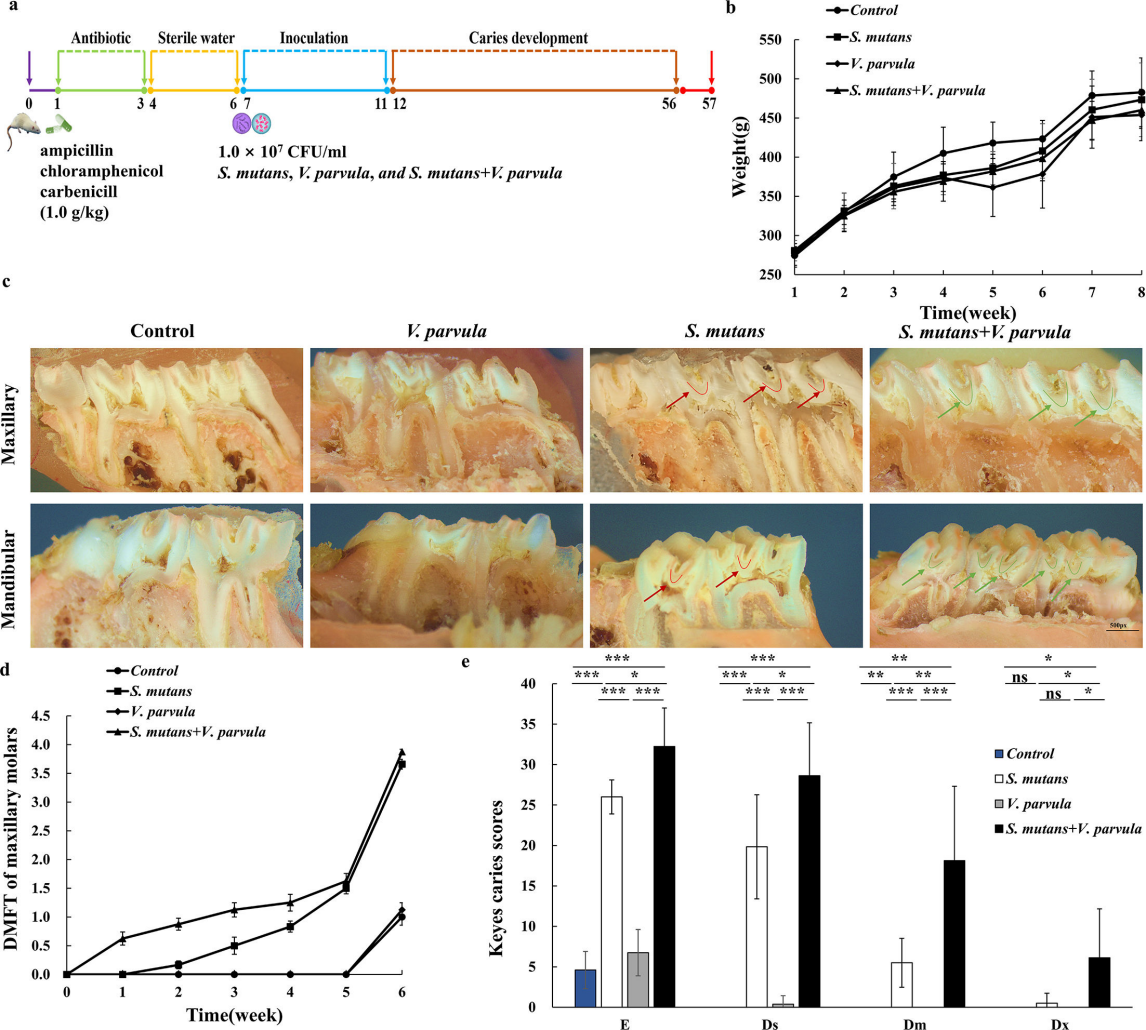
Investigation the biofilm virulence of *S. mutans* and *V. parvula in vivo*. (**a**) Design and construction of caries induced animal model. (**b**) Body weights of rats during the experimental period. (**c**) Images of caries lesions on the rat maxillary and mandibular teeth. Red arrow, lesions represent the involvement of 1/4–3/4 of the dentin region; Green arrow, lesions represent caries progression beyond 3/4 of the dentin region. (**d**) Rate of caries in maxillary molars. (**e**) Caries onset and severity of sulcal surfaces on the maxillary and mandibular teeth. Caries scores are recorded as stages and extent of carious lesion severity according to Keyes’ scoring system. The Keyes scoring method divided the molar into several units according to the range of caries. E: enamel infection; Ds: 0–1/4 of the dentin region; Dm: 1/4–3/4 of the dentine infection; Dx: beyond 3/4 of the dentin region. The experiments were performed in biological and technical triplicates. Data were presented as the mean ± SDs. *P < 0.05, **P < 0.01, and ***P < 0.001; ns, nonsignificant.

To validate the *in vivo* cariogenicity of the dual-species biofilm, we preestablished a cariogenic rodent model according to the plan shown in Fig. 5a. All animals remained in good health until the experimental endpoint and gained weight equally without significant differences among groups (*P* > 0.05, Fig. 5b). In order to observe the occurrence and progression of caries in each group, the rate of caries occurrence in maxillary molars was recorded (Fig. 5d). At the same time, the molar incisions of rats treated with 0.06% mureoxide staining were photographed to observe the degree of decay. Minor tooth demineralization with no severe caries lesions occurred in the control or *V. parvula* group. And, demineralization only occurred at the end of the experiment, and the rate of caries was slower. Compared with *S. mutans* group, in which dental caries appeared in maxillary molars two weeks after inoculation, caries in *S. mutans* and *V. parvula* co-inoculated group appeared in maxillary molars one week after inoculation, and the rate of caries was higher than that in *S. mutans* group throughout the observation period (*P* < 0.01)(Fig. 5d). Photographs of the caries lesions (Fig. 5c) on the maxillary and mandibular teeth of the rats were obtained using the stereo microscope. The results of Keyes’ scores differed in how much and how severely different interventions caused sulcal surfaces of molars (Fig. 5e). As expected, the corresponding caries in the *V. parvula* group were limited to enamel demineralization and relatively fewer superficial dentin demineralization, which displayed no significant differences from the control group, suggesting that *V. parvula* alone has little cariogenic potential. However, *S. mutans* co-infected with *V. parvula* resulted in a significant increase in both caries progression, total caries and the severity of caries lesions compared to those infected with species alone, indicating enhanced virulence within symbiotic microbiota. Notably, we found that co-infection produced more severe lesions, characterized by more extensive dentinal depth destruction. In sum, we revealed that the interaction between *V. parvula* and *S. mutans* enhanced the biofilm structure, and the presence of *V. parvula* leads to the increased virulence of *S. mutans* that caused the severe dental caries *in vivo*, suggesting that the interspecies relationship promoted the disease-causing conditions.

## DISCUSSION

In the current study, we first investigated the dental plaque microbiome profiles in ASC and used conservative selection criteria to identify bacterial species associated with disease. *V. parvula* was identified as strongly associated with ASC and could be used as an early warning biomarker of ASC. *V. parvula* and other oral *Veillonella* spp. are early oral bridging colonizers and metabolize the lactic acid produced by *Streptococci* to establish early multispecies biofilm communities on hard tooth surfaces (22). Recent research revealed that *Veillonella* species have clear, differential site specificity: *V. parvula* showed strong preference for supra- and subgingival plaque. The “site-special” helps it adhere onto tissues (mostly non-shedding dental tissues) at sites that best cover their physiological needs, named their preferred ecological niche. A cross-sectional study found that the levels and distribution of *S. mutans* in the posterior teeth at different dentition stages were associated with the corresponding tooth-specific microbiome including *Veillonella* spp. (23). This site specificty and coaggregation interactions of *Veillonella* spp. with early oral colonizers may enhance the recruitment of a highly adaptable strain to a given microbial community, which may thereby foster the establishment of more pathogenic species. And the metabolism of *Veillonella* spp. may alter their immediate environment and thereby foster the oral microecological imbalance. For instance, their catalase activity can create favorable low redox potential conditions for growth by more oxygen-sensitive anaerobes (*Fusobacterium nucleatum*), as would be periodontal pathogens in the gingival sulcus (24). *Veillonella* spp. was also shown to produce heme to support *P. gingivalis* growth *in vitro* (25), which suggests that *Veillonella* spp. could be a potential hemin/heme provider to support periodontopathogen growth and might play a crucial role in dental biofilm formation and periodontitis development. Recent clinical studies on root caries, ECC, SECC, and stunted children have found that the detection rate and abundance of *V. parvula* in caries group are higher than that in caries-free group, which may be an important factor leading to oral microecological imbalance in caries (26–29). It also may be purported that the high abundance of *V. parvula* stems from their metabolic requirement for organic acids that are, indeed, found in higher concentrations in active caries. *V. parvula* was considered an ecological beneficiaries of an incipient dysbiotic community. Indeed, by establishing an artificial mouth model using human enamel slabs, Noorda et al. (30) found evidence of an increased acid production of *S. mutans* co-cultured with *Veillonella alcalescens*. Co-cultures are also found in resulting in higher enamel surface demineralization under anaerobic conditions. Those consistent observations indicated that the non-negligible role of *Veillonella* spp. in correlation with caries progression (31–34). Based on these researches, *Veillonella* spp. haven been recently proposed to behave as “accessory pathogens” and considered playing an important role in the ecology of oral biofilms and the development of caries (24, 35). The role and mechanism of *Veillonella* spp. in the pathogenesis of caries, nonetheless, remains unconfirmed. Hence, the interaction of *V. parvula* and *S. mutans* was subjected into further comprehensive biofilm studies, which may provide further insights on the pathogenic mechanisms in ASC. In addition, there are also some limitations about our study. The sample size limited the statistical power, especially a low number of patients (seven individuals in the high caries group) and healthy individuals (six in the caries-free group), which may have accounted for the absence of significant differences in the microbiome α-diversity. Even so, we also think the findings would give some significant inspiration for the research because some differences were already seen at a small statistical power.

Another key finding of the study is that *V. parvula* can exacerbate *S. mutans* virulence. Lactate, the secondary metabolites of *S. mutans*, has been the preferred carbon source for most *Veillonella* species (35). The metabolic consumption of lactate establishes a food chain relationship between *Veillonella* spp. and *S. mutans.* Some scholars believe that lactate is the strongest acid produced in significant amount by oral taxa and is involved in the demineralization of dental hard tissues. Its conversion to weaker volatile acids by *Veillonella* spp. may, therefore, improve the low pH environment caused by *S. mutans* acting as a buffer to the carious damages induced by *S. mutans.* But the pH dynamic curve showed that *V. parvula* could promote acid production of *S. mutans*. As is known to all, the acid tolerance of *S. mutans* can help itself survive a lethal acidified microenvironment (36). Another key finding of the present report is that co-culturing with *V. parvula* significantly enhanced the acid resistance of *S. mutans* (Fig. 2B). The *V. parvula* metabolism of lactate may allow *S. mutans* to grow to higher levels before reaching pH values that inhibit further bacterial growth and affect the viability of *S. mutans*. Bradshaw et al. (37) found that when pH dropped below 4.5, the growth of bacteria lacking acid resistance was inhibited, whereas *V. parvula* and some cariogenic bacteria became more competitive and became the dominant bacteria. In addition, the distribution of *V. parvula* and *S. mutans* was highly correlated. Though *V. parvula* was classified as strict anaerobes, its catalase activity was shown to detoxify ambient hydrogen peroxide (H_2_O_2_). For instance, co-culturing with *V. parvula* can reduce the inhibition of H_2_O_2_ on *S. mutans* biofilm (38). Zhou et al. (24) integrated the catalase gene (*catA*) of *V. parvula* PK1910 into *V. atypica* OK5, confirming that *catA* gene products can protect the growth of other bacteria such as *F. nucleatum* in the H_2_O_2_ environment produced by *S. gordonii*. The hydrogen peroxide killing assay in our study also confirmed the ability of *V. parvula* to detoxify hydrogen peroxide on *S. mutans*. So, the unique metabolism of *V. parvula* may alter its immediate environment and thereby foster the establishment of more pathogenic species.

A lot of studies (39–41) have repeatedly verified by means of the dual-species biological model that *Veillonella* spp. can promote the formation of *S. mutans* biofilm. When being co-cultured with *Veillonella* spp., the number of *S. mutans* in the dual-species biofilm increased compared with that cultured alone. This promotion effect still existed in the multi-strains biofilm. The synergy in the formation of biofilm is closely related to the adhesion among bacteria. Kara et al. (41) found that the spatial allocation of *S. mutans* and *V. parvula* in the dual-strain biofilm was less than 1.2 µm. In this study, we recognized that *V. parvula* cannot adhere to hydroxyapatite slides to form biofilm but can construct a stable and powerful biofilm structure by adhering and aggregating to the long chain structure of *S. mutans*. This suggested that the adhesion of *S. mutans* to hydroxyapatite slides may have provided a site for the adhesion of *V. parvula*. This dependence on *S. mutans* to provide sites for adhesion growth and formation of specific spatial adhesion structure has also been demonstrated in the oral environment of saliva flow (42). Hag1 was the first surface protein to have been identified in *V. atypica* OK5 strain, and the encoding of *hag1* gene is required for its coaggregation with *S.gordonii*, *Streptococcus oralis*, *Streptococcus cristatus*, *Porphyromonas gingivalis* and even for adhesion to human oral epithelial cells (43). The coaggregation of *Veillonella* spp. and *S. mutans* also contributed to the growth of *S. mutans* in the biofilm. But the coaggregation with different partners is likely to involve distinct domains and mechanisms, warranting further investigation. Hence, the enhanced level of mixed biofilm with increased bacteria we observed in crystal violet assay and live/dead bacterial staining in this study mainly concerned with the adhesion and co-aggregation of *S. mutans* and *V. parvula*. Moreover, EPS quantification and CLSM analysis revealed that the enhanced level of dual-strain biofilm was partly attributed to increased production of EPS. EPS is the main component of the extracellular matrix of bacterial biofilms, and the ability to synthesize EPS is a vital virulence factor of *S. mutans*, the roles of which are closely related to the *S. mutans* GTF system (21, 44, 45). Co-culture dramatically increased the gene *gtfB*, *gtfC*, and *gtfD* expression of the *S. mutans* in dual-species biofilm (Fig. 4b). And the EPS synthesis demonstrated more production of EPS in mixed biofilms versus *S. mutans* alone (Fig. 4a). EPS can stabilize biofilms in a compact and rigid structure and further aggravate the acidic cariogenic microenvironment. Additionally, glucans can provide binding sites for *S. mutans* and other oral microorganisms to help them further colonise and aggregate (46). In general, our study corroborated that *V. parvula* can effectively promote *S. mutans* mature biofilm formation with increased bacteria and extracellular matrix and is mainly attributed to the boost of the adhesion, coaggregation growth, and EPS synthesis of *S. mutans*.

Considering that the biofilm spatial structuring of *S. mutans* and *V. parvula* may create a virulent cariogenic microenvironment, we investigated the biofilm virulence of *S. mutans* and *V. parvula* using a rodent model. As predicted, though incapable of causing disease on its own, *V. parvula* significantly heightened the biofilm virulence of *S. mutans in vivo* when co-infecting and augmenting the progression, quantity, and severity of dental caries, which suggested the possibility that *Veillonella* species may act as “accessory pathogens,” having the capabilities to promote the growth of *S. mutans* within oral biofilms, finally inducing dental caries. *Veillonella* spp. has so far been implicated in dental caries, endodontic infections, periapical disease, periodontal disease, peri-implantitis, halitosis, and other oral diseases (27, 47–50), but the specific mechanism of its influence on oral diseases at the molecular level still need further studies. Our findings demonstrate that *V. parvula* acts as a pathobiont to modulate the metabolic activity of *S. mutans*, spatial structure, and pathogenicity of biofilms of *S. mutans* in the context of ASC.

## MATERIALS AND METHODS

### Clinical sample analyses

Seven patients diagnosed with ASC were recruited from the Department of Endodontics, Nanjing Stomatological Hospital, Affiliated Hospital of Medical School, Nanjing University from October 2022 to April 2023 as the observed group (high caries, HC group). In addition, six healthy individuals without caries who met the criteria were selected from the doctors and medical students in the same hospital as the control group (caries-free, CF group). Ethical approval was obtained from the Ethical Committee (Ethics NO, NJSH-2022NL-47). The study has been registered in the clinical registry with a registration number “ChiCTR1800017220.” All participants signed informed consent forms. The inclusion criteria were according to previous studies (6) as follows: (i) patients over the age of 18 and less than 40 years of age; (ii) patients with no obvious periodontal abscesses, precancerous lesions, fungal infections, peri-crown of wisdom teeth inflammatory, or any other oral diseases; (iii) the quantity of remaining teeth in the mouth more than 24 for each participants; and (iv) HC group: with decayed, missing, or filled teeth (DMFT) ≥9; at least eight open coronal carious cavities and one anterior tooth caries; the score of decayed, missing, or filled surfaces (DMFS) ≥18. CF group: DMFT = 0; DMFS = 0. The exclusion criteria were set as follows: (i) patients who had received oral medication or surgery in the past 6 months; (ii) patients who had received antibiotics, hormones, or immunosuppressants systemic in the past 6 months; (iii) patients with systemic disease like Sjogren syndrome, psychiatric disorders, pregnancy or lactation, or any other oral diseases that might affect the oral status; (iv) patients receiving radiotherapy; and (v) smoking patients. All the included patients were confirmed by an experienced endodontist. A total of 13 eligible patients were recruited for contribution of the present study.

All participants abstained from eating, drinking, brushing, and any oral hygiene the night before sampling until the morning of day 2 for dental plaque sample collection. After being grouped, all collected samples were frozen in liquid nitrogen and then stored at −80°C until use.

### Bioinformatic analysis

The Metagenome sequencing and analysis were conducted by OE Biotech Co., Ltd. (Shanghai, China). The taxonomy of the species was obtained as a result of the corresponding taxonomy database of the NR Library, and the abundance of the species was calculated using the corresponding abundance of the genes. In order to construct the abundance profile on the corresponding taxonomy level, abundance statistics were performed at each level of Domain, Kingdom, Phylum, Class, Order, Family, Genus, and Species. The PCA analysis and plotting of the taxonomy abundance spectrum or functional abundance spectrum were carried out using Rsoftware (v 3.2.0), and the results of the equidistant matrix of PCoA and NMDS were calculated and analyzed. Then, the R package was used to analyze the significant differences between different groups using *T* test statistical test. The liner discriminant analysis effect size (LEFSe) method was used to compare the taxonomy abundance spectrum or functional abundance spectrum.

### Bacterial strains and growth conditions

*Streptococcus mutans* BNCC 337082 and *Veillonella parvula* BNCC 294853 were obtained from the BeNa Culture Collection (Henan, China) and used in this study. *S. mutans* was routinely anaerobically (85% N_2_, 5% CO_2_, 10% H_2_) incubated at 37°C in brain heart infusion (BHI) broth (Difco, MD, USA) or on BHI agar plates. *V. parvula* cultures and agar plates were grown at 37°C in the same anaerobic conditions using BHI medium supplemented with 0.6% sodium lactate (Macklin, China). For the biofilm studies, 1% sucrose was added to the BHI broth. Mono- and dual-species biofilms were grown on saliva-coated hydroxyapatite slides placed in 24-well plates horizontally with 2.0 mL of microbial suspensions at 37°C for 24 h under facultative anaerobic conditions without disturbance.

### Glycolytic PH drop, acid killing, and hydrogen peroxide challenge

Bacteria were harvested at mid-logarithmic phase by centrifugation (4,500 rpm, 4°C, 15 min), washed with phosphate-buffered saline (pH 6.5), and re-suspended in the same solution (OD_600_ ≈ 0.5). And the pH values of both single- and dual-species were detected over a period of 210 min with an electronic pH meter.

The ability of *S. mutans* to withstand acid stress or oxidative stress cultivating with *V. parvula* was determined by modification of a previously published method (51). Briefly, bacteria were grown in medium adjusted to pH 7.0 with HCl to mid-exponential phase (OD_600_ ≈ 0.5), harvested by centrifugation at 4,500 rpm at 4°C for 15 min and washed with an equal volume 0.1 M glycine buffer (pH 7.0). For acid-killing assays, cells were incubated in 5 mL 0.1 M glycine buffer (pH 2.8) for 15, 30, or 45 min. For oxidative stress tolerance assays, cells were re-suspended in 5 mL 0.1 M glycine buffer (pH 7.0), and hydrogen peroxide (H_2_O_2_, South China, China) was then added to give a final concentration of 0.2% (vol/vol) for 15, 30, or 45 min, and the survival rate was determined by plating in triplicate on BHI plates.

### Biofilm SEM scanning and biomass quantification

Bioﬁlms were harvested and fixed with glutaraldehyde (2.5%) overnight at 4°C, washed three times with PBS, dehydrated using a series of ethanol rinses (30%, 50%, 70%, 80%, 85%, 90%, 95%, and 100%), immersed for 10 min in 100% ethanol, and dried in a desiccator. After coating with gold-palladium, specimens were examined in a scanning electron microscope (S-3400N; Hitachi, Tokyo, Japan) at 300×, 1,000×, 3,000×, 10,000× magnification. For the biofilm formation assay, the biofilms formed in 96-well microtiter plates were fixed with 4% paraformaldehyde for 30 min, washed three times with PBS, stained with 0.5% crystal violet (Sigma-Aldrich, CA, USA) for 30 min, and washed three times with PBS. The crystal violet was extracted with 200 µL of 100% ethanol. The extract was evaluated at 600 nm using a microplate reader (Molecular Devices, CA, USA).

### Live/dead bacteria staining and EPS staining

The biofilms were stained using the BacLight live/dead bacterial viability kit (Molecular Probes Inc, OR, USA). Viable cells and dead cells were, respectively, stained with SYTO 9 (excitation, 480 nm; emission, 500 nm) and propidium iodide (PI; excitation, 490 nm; emission, 635 nm). Samples were observed with confocal laser scanning microscopy (CLSM; NikonTi; Nikon Inc., Tokyo, Japan). Images were taken at an interval of 5 µm. Three-dimensional reconstruction of the biofilms and integrated ﬂuorescence density was quantiﬁed with software NIS-Elements AR (NikonTi; Nikon Inc., Tokyo, Japan). An EPS assay was conducted according to previous studies (52). In brief, the biofilms cultured on hydroxyapatite slides were grown in BHIS medium and supplemented with 1 µM Alexa 647 red fluorescent dye (Molecular Probes Inc., OR, USA) and protected from light. After incubation for 24 h, the biofilms were incubated with 1 µM SYTO 9 green fluorescent dye (Molecular Probes Inc., OR, USA) at room temperature for 20 min. Alexa Fluor 647 (excitation, 650 nm; emission, 668 nm) was used to label EPS, and SYTO 9 was used to label bacterial cells. Samples were then observed using CLSM with the same procedure as live/dead staining.

### qRT-PCR analysis of *gtf* gene

Quantitative RT-PCR was used to quantify the expression of *gtfB, gtfC,* and *gtfD* genes, with *16S rRNA* as an internal control. Bacteria RNA was extracted using the RNAprep Pure Tissue Kit (Tiangen, Beijing, China) and then reverse transcribed into cDNA using the FastKing RT Kit (Tiangen, Beijing, China). An Talent qPCR PreMix kit (Tiangen, Beijing, China) was used for quantitative polymerase chain reaction (qPCR) following the manufacturer’s instructions. All reactions were carried out in triplicate. Analysis was performed using the 2^−ΔΔCt^ method. The primers used in this study are listed in Table 2.

**TABLE 2:** Specific primers for the *S. mutans* virulence-associated genes

Genes	Primer sequence (Forward and Reverse)	Amplicon size
*16S rDNA*	F: AGCGTTGTCCGGATTTATTG R: CTACGCATTTCACCGCTACA	156 bp
*gtfB*	F: CACTATCGGCGGTTACGAAT R: CAATTTGGAGCAAGTCAGCA	194 bp
*gtfC*	F: GATGCTGCAAACTTCGAACA R: TATTGACGCTGCGTTTCTTG	164 bp
*gtfD*	F: TTGACGGTGTTCGTGTTGAT R: AAAGCGATAGGCGCAGTTTA	219 bp

### *In vivo* models of dental caries

The experimental animal protocol was approved by the ethics authorities of Nanjing University (IACUC-D2203007), and all experiments were performed according to the National Institutes of Health Guide for the Care and Use of Laboratory Animals. Twenty-seven pathogen-free (SPF) male SD rats, aged 35 days, were randomly divided into four groups: control (*n* = 5), *S. mutans* (*n* = 6), *V. parvula* (*n* = 8), and *S. mutans + V. parvula* (*n* = 8). Upon arrival, animals were determined to be free of any indigenous oral microorganism by feeding the rats antibiotics, namely, ampicillin, chloramphenicol, and carbenicillin (1.0 g/kg), for three consecutive days. Then saliva from rats was inoculated on Mitis Salivarius-bactericin Agar (MSBA) containing 0.2 µg/mL Bacillus for 48 h to check the growth of *S. mutans.* After a washout period of 3 days, in which the residual antibiotics in the oral cavity were removed by feeding rats antibiotic-free distilled water, rats were challenged with 1.0 × 10^7^ CFU/mL *S. mutans*, *V. parvula*, or *S. mutans + V. parvula* suspensions for five consecutive days (once per day with no food or water for 2 h after incubation). The bacteria were inoculated on both molar teeth of rats with sterile cotton swabs. All rats were fed the National Institutes of Health cariogenic diet 2000 and 5% sucrose water. The experiment lasted for 2 months. The occurrence of caries in each group was recorded, and then the rats were sacrificed. The jaws of the rats were aseptically dissected, and the caries status was scored using the Keyes method.

### Statistical analysis

The statistical analyses were carried out with GraphPad Prism 9 unless otherwise specified. Differences between two groups were evaluated using a two-tailed Student’s *t*-test (parametric), the Mann-Whitney *U* test (nonparametric), or Fisher’s precision probability test. For more than two groups, one-way analysis of variance (ANOVA; parametric) or the Kruskal–Wallis test (nonparametric) was performed, followed by Bonferroni’s multiple comparisons test. A value of *P* < 0.05 was considered significantly significant.

## Data Availability

The data presented in the study are deposited in the NCBI SRA database, accession number PRJNA1002887.

## CLINICAL TRIAL REGISTRATION

The study has been registered in the clinical registry with a registration number "ChiCTR1800017220." All participants signed informed consent forms.
